# Supplementary material for: WATCH-SS: Developing a Trustworthy and Explainable Modular Framework for Detecting Cognitive Impairment from Spontaneous Speech
Source: medRxiv. 2025 Oct 28:2025.08.06.25333047. Preprint. [Version 2] doi: 10.1101/2025.08.06.25333047 (PMC12636657; doi:10.1101/2025.08.06.25333047)
Supplement: Supplement 1 [file media-1.pdf]

WATCH-SS: Developing a Trustworthy and Explainable Modular Framework for Detecting Cognitive Impairment from Spontaneous Speech*

Sydney Pugh, PhD[1†], Matthew Hill[1], Sy Hwang, MS[1], Rachel Wu[1], Kuk Jang, PhD[4], Stacy Iannone, DHSc, MS[2], Karen O'Connor, MS[1], Kyra O'Brien, MD, MSHP[2], Eric Eaton, PhD[3], and Kevin Johnson, MD, MS[1,3]

[1] *Department of Biostatistics, Epidemiology, and Informatics, Perelman School of Medicine, University of Pennsylvania, Philadelphia, PA USA*

[2] *Department of Neurology, Perelman School of Medicine, University of Pennsylvania, Philadelphia, Pennsylvania, USA*

[3] *Department of Computer and Information Science, School of Engineering and Applied Science, University of Pennsylvania, Philadelphia, PA USA*

[4] *Department of Computer Engineering, Hongik University, Seoul, South Korea*

[†] *Corresponding Author E-mail: Sydney.Pugh@PennMedicine.upenn.edu*

Early detection of cognitive impairment (CI) is critical for timely intervention in Alzheimer's disease and AD-related dementias. To address this, we propose the Warning Assessment and Alerting Tool for Cognitive Health from Spontaneous Speech (WATCH-SS), a modular and explainable three-stage framework for detecting CI from a patient's speech sample. The framework uses detectors for five linguistic and acoustic indicators of CI, aggregates their outputs into a set of clinically interpretable summary features, and uses a predictive model for CI classification. We consider multiple approaches to implementing these detectors that range from simple, computationally efficient methods suitable for real-time analysis to strong, resource-intensive methods, better for high accuracy offline analysis. On the DementiaBank ADReSS dataset, WATCH-SS achieved strong predictive performance (AUC = 80% on the test set). This work demonstrates that a modular, feature-based approach can achieve strong performance while providing a transparent diagnostic profile, representing a significant step towards a trustworthy and clinically-usable screening tool for primary care.

Keywords: cognitive impairment, Alzheimer's disease, dementia, natural language processing, large language model, machine learning

*Research reported in this publication was supported by the National Institute On Aging of the National Institutes of Health under Award Number P30AG073105, and by the University of Pennsylvania ASSET Center under the Collaborative Research in Trustworthy AI for Medicine grant. The content is solely the responsibility of the authors and does not necessarily represent the official views of the National Institutes of Health.

Preprint of an article submitted for consideration in Pacific Symposium on Biocomputing © 2025 World Scientific Publishing Co., Singapore, http://psb.stanford.edu/.

1. Introduction

As the global population ages, Alzheimer's disease (AD) and AD-related dementias (ADRD) are becoming more prevalent.[1] Yet, more than 50% of persons with AD/ADRD are undiagnosed,[2] delaying crucial interventions that could improve quality of life. The first step in diagnosing AD/ADRD is detecting any degree of cognitive impairment (CI). Ideally, early detection should occur in primary care, where most patients first exhibit subtle signs of CI. Language production is highly sensitive to the neurodegenerative changes characteristic of AD/ADRD. Deficits in core cognitive domains—including semantic memory, executive function, and processing speed—manifest directly in a person's speech patterns.[3–5] Several of these signs manifest in conversations between patients and primary care providers (PCPs) during clinical visits—for example, when a patient repeats themselves several times during the visit or takes a long time to respond to the PCP's questions. However, PCPs often struggle to recognize these clues in real time due to competing priorities, time constraints, and limited expertise.[6]

Speech data can be collected easily and non-invasively, making it an ideal medium for developing accessible tools for early-stage screening of CI. Recent advances in machine learning and natural language processing (NLP) have enabled the development of automated systems for detecting possible CI from speech.[7–9] However, many state-of-the-art approaches rely on "black-box" deep learning models.[10–12] While these systems can achieve high predictive accuracy, their opaque nature makes it impossible to understand the clinical reasoning behind a prediction, hindering trust and adoption in medical settings.

To address this gap, we propose the Warning Assessment and Alerting Tool for Cognitive Health for Spontaneous Speech (WATCH-SS), a modular framework designed to be a trustworthy, interpretable, and extensible tool for detecting CI due to suspected AD/ADRD from a patient's speech sample. The framework leverages several detectors to identify indicators of CI within a speech sample and then aggregates the outputs into a clinically interpretable CI prediction. We demonstrate that our framework achieves strong predictive performance while providing a transparent diagnostic profile that explains the reasoning behind its predictions.

2. Related Work

Spontaneous speech, reliant on cognitive functions like attention and memory, is increasingly used as a non-invasive, cost-effective biomarker for detecting cognitive impairments. Clinically, speech is typically analyzed via structured speech tasks like the *Cookie Theft Picture Description* task from the Boston Diagnostic Aphasia Examination,[13] verbal fluency tasks such as the Semantic Fluency Task (SFT) and Phonemic Fluency Task (PFT),[14] and cognitive tests including the Mini-Mental State Examination (MMSE),[15] the Montreal Cognitive Assessment (MoCA),[16] and the Saint Louis University Mental Status (SLUMS) examination.[17] While these assessments have been rigorously validated, they typically require in-person administration and expert interpretation, making them time-consuming and costly, which can delay diagnosis and treatment of neurodegenerative conditions like AD/ADRD.

Advances in machine learning offer the potential to overcome these limitations by enabling low-cost, remote, and early detection of cognitive decline through speech. They have revealed subtle linguistic and acoustic markers, expanded clinically relevant feature extraction, and

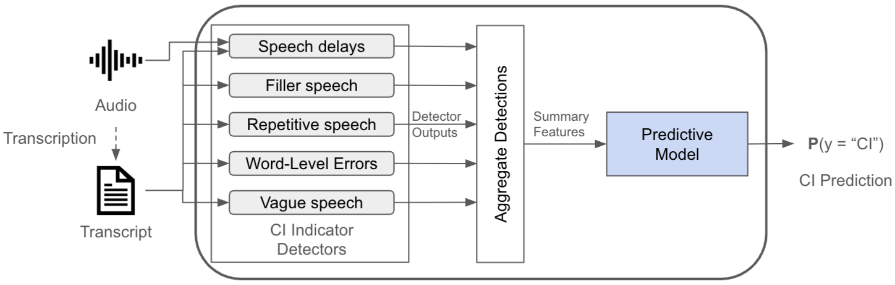

Fig. 1: The WATCH-SS framework is a three-stage pipeline that (1) detects cognitive impairment (CI) indicators from audio and transcripts, (2) aggregates these detections into summary features, and (3) applies a predictive model to the features to generate a final prediction.

enabled scalable, automated speech analysis.

To address these limitations, machine learning has been widely applied to automate the analysis of speech for detecting CI. Early works focused on training traditional models like Support Vector Machines (SVMs) on hand-crafted acoustic features (e.g., pause duration, speech rate)[18] and lexical features (e.g., topic modeling).[19] We refer the reader to Yang et al. [20] for an extensive survey of deep learning-based techniques. More recently, deep learning approaches using models like Convolutional Neural Networks (CNNs) and Long Short-Term Memory networks (LSTMs) have been used to learn patterns directly from audio.[21–23] Transfer learning with large pre-trained models such as BERT, GPT, and wav2vec has also become a common strategy for extracting powerful text and audio embeddings for training various ML learning classifiers.[21,24–26] These methods, often combined in multimodal or ensemble systems,[27,28] have demonstrated high predictive accuracy but low explainability.

While other approaches have also explored training classifiers on interpretable speech features,[8,13,29,30] they often use a mix of clinically interpretable and non-interpretable features. For example, these systems often incorporate acoustic features like GeMAPS or ComParE which are not easily parsed by clinicians. In contrast, WATCH-SS is designed to provide greater interpretability by focusing on high-level, clinician-interpretable features. This approach, coupled with external validation on spontaneous clinical speech, differentiates our work.

3. Methods

3.1. *WATCH-SS*

We propose WATCH-SS, a three-stage framework for detecting cognitive impairment from a patient's speech sample (see Figure 1). The key advantages of our framework are its interpretability and modularity. Unlike "black-box" models, WATCH-SS provides a transparent patient diagnostic profile that breaks down the final CI prediction into the contributions of various indicators of CI, enabling a clinician to understand which specific cognitive deficits are driving the classification. Moreover, the modular architecture of WATCH-SS allows for easy extension or replacement of individual detectors.

WATCH-SS receives as input an audio waveform and the corresponding diarized transcript of a speech sample. First, WATCH-SS runs multiple detectors in parallel to identify five

key indicators of CI: filler speech, repetitive speech, substitution errors, vague speech, and speech delays. In the second stage, the system aggregates the detections into a set of clinically interpretable summary features and feeds them to a model to predict CI in the third stage.

WATCH-SS was implemented in Python (version 3.12.3) using Microsoft Azure Databricks. The code is available at https://github.com/kbjohnson-penn/WATCH-SS.

The remainder of this section describes the data used to develop WATCH-SS (Section 3.2), the detectors for indicators of CI (Section 3.3), the summary features extracted from these detections (Section 3.4), and finally, the predictive model for CI (Section 3.5).

3.2. *Data*

We developed and validated WATCH-SS using two datasets: the ADReSS dataset for detector and predictive model development, and the OBSERVER dataset for external validation.

3.2.1. *DementiaBank ADReSS Challenge Dataset*

The ADReSS dataset[31,32] consists of audio recordings and diarized transcripts of subjects with and without an AD diagnosis describing the Cookie Theft picture from the Boston Diagnostic Aphasia Exam.[33] Transcripts were annotated using the CHAT coding system, a standardized protocol for transcribing conversational interactions and annotating its linguistic features.[34] The training data consists of 108 subjects, while the test data consists of 48 subjects. Each dataset is balanced in age and gender and half of their subjects have an AD diagnosis.

We further partitioned the training data into training and development (dev) datasets using a 70/30 percent split balanced for age, gender, and number of AD subjects. Specifically, we first generated 100 candidate splits stratified by gender and AD label using different random seeds. Then, we selected the split that minimized the absolute difference in mean age across the resulting training and development sets. Table 1 summarizes the resulting datasets.

Data Pre-processing. We processed the CHAT transcripts to produce clean inputs to our detectors by: (1) converting pause annotations to a "[silence]" token, (2) converting event annotations to "[< event >]" tokens, (3) converting inaudible annotations to "[inaudible]" tokens, and (4) removing all other CHAT annotations. Then, we derived ground-truth labels from the original CHAT annotations for all patient utterances. An utterance was labeled positive for a given indicator if it met the following criteria:

- **Filler speech:** The utterance contained any word marked with the filler prefix ("&").
- **Repetitive speech:** The utterance contained the repetition annotation ("[/]").
- **Substitution errors:** The utterance contained the word level error code ("[*]"), including all its subtypes (*e.g.,* "[* s]" for semantic paraphasias or "[* n]" for neologisms).
- **Vague speech:** The utterance contained the annotation for empty speech ("[+ es]") or circumlocution ("[+ cir]").
- **Speech delays:** The utterance contained a pause annotation ("(.)", "(..)", or "(...)").

The prevalence of these labels across the data splits is summarized in Table 2.

Table 1: Characteristics of the ADReSS train, development (dev), and test datasets.

		AD		Control	
Split	Num. Subjects	Pct. Female	Age (mean ± std)	Pct. Female	Age (mean ± std)
train	75	56%	66.5 ± 6.6	55%	66.6 ± 6.8
dev	33	52%	67.4 ± 6.7	56%	65.7 ± 6.0
test	48	54%	66.1 ± 7.4	54%	66.1 ± 7.1

Table 2: Prevalence of cognitive impairment (CI) indicators in the ADReSS train, development (dev), and test datasets. Values are the counts and proportions of positively-labeled utterances.

Split	Num. Utterances	Filler speech	Repetitive speech	Substitution errors	Vague speech	Speech delays
train	991	257 (18.4%)	88 (6.3%)	35 (2.5%)	12 (0.9%)	99 (7.1%)
dev	501	118 (16.6%)	44 (6.2%)	19 (2.7%)	3 (0.4%)	45 (6.3%)
test	590	153 (18.9%)	47 (5.8%)	25 (3.1%)	8 (1.0%)	98 (12.1%)

Table 3: Characteristics of the OBSERVER Repository dataset.

CI			CN		
Num. Visits	Pct. Female	Age (mean ± std)	Num. Visits	Pct. Female	Age (mean ± std)
12	50%	75.0 ± 10.1	12	50%	74.1 ± 8.6

3.2.2. *Penn OBSERVER Repository Dataset*

The OBSERVER Repository was developed by the University of Pennsylvania to capture and support detailed, multimodal analyses of real outpatient clinical visits.[35] For this work, we used the visit recordings, diarized manual transcripts, and corresponding Electronic Health Record (EHR) data from the repository. We preprocess the audio from the recordings to reduce any background noise using Adobe Premiere Pro's audio enhancement tools. [a]

To construct our external validation dataset, we first selected the earliest recorded visit for all patients aged 50 or older (74 patients in total). Next, to identify patients with CI we reviewed each patient's EHR data available up to the time of visit for mention of “cognitive impairment”, “Alzheimer's”, or “dementia” in the diagnosis list, problem list, medical history, or encounter notes. This process identified 14 CI patients, two of which we excluded because their CI was not due to suspected AD/ADRD. To create a balanced case-control dataset, we downsampled the cognitively normal (“CN”) pool to 12 patients that best balances age and gender with the CI group. Table 3 summarizes the dataset.

[a]https://www.adobe.com/products/premiere.html

3.3. *Detectors for Indicators of Cognitive Impairment*

We developed detectors for five key indicators of CI. Four are linguistic indicators extracted from the transcript–filler speech, repetitive speech, substitution errors, and vague speech–whereas the fifth indicator, speech delays, is an acoustic indicator derived from the audio.

A significant challenge to developing specialized detectors for these CI indicators is the lack of large, publicly available annotated datasets. To overcome this, we focus on low-cost approaches that are straightforward to implement and do not require extensive amounts of annotated data for task-specific fine-tuning. We implemented a single detector for speech delays, as their identification relies on silence detection–a well-established audio signal processing task. For the linguistic indicators, however, we implement and compare two primary approaches:

- **Traditional NLP**: Computationally efficient methods for capturing well-defined linguistic patterns that serve as strong, high-recall baselines.
- **LLM Prompting**: To evaluate the "out-of-the-box" capability of modern, pre-trained LLMs for detecting linguistic indicators of CI we use zero- and few-shot prompting.

To implement the LLM-based detectors, we created a general-purpose prompt template, shown in Figure 2, that we adapt for each specific CI indicator. The prompt instructs the LLM to adopt the persona of a neurologist, provides a detailed definition of the target CI indicator with rules for detection, and enforces a JSON output format. We iteratively refined all prompts for linguistic indicators to maximize the F1-score on the ADReSS training dataset. For the few-shot prompts, examples were selected from the ADReSS development dataset based on an error analysis of the zero-shot version of the prompt. All detectors use OpenAI's GPT-4o LLM [b], which has shown promising performance on clinical tasks like speech analysis.[36–38] To ensure reproducibility and reduce non-determinism, we fix the LLM's *temperature* to 0 and *top_p* to 1.

These approaches offer different balances between detection accuracy and computational efficiency, allowing WATCH-SS to be configured with either fast, lightweight detectors for real-time screening or more computationally intensive, high-accuracy detectors for offline analysis. The following subsections detail the clinical motivation for each indicator and the specific implementation of these detector types.

3.3.1. *Filler Speech*

Fillers are sounds, words, or phrases used to improve speech planning, often as an alternative to silent pauses. Frequent use of fillers is a well-documented linguistic indicator of word-finding difficulties and increased cognitive load associated with CI and AD.[5]

NLP Baseline: Keyword Search Detector. Our baseline for filler speech is a simple, case-insensitive keyword search that processes each utterance and returns the text and character span for any matches. We use the spaCy Python library (version 3.8.7),[39] with the pre-trained English model pipeline optimized for CPU "en_core_web_md", for tokenization and keyword matching. To determine the optimal filler keyword set, we evaluated combinations of the

[b] Experiments involving LLMs were conducted using HIPAA-compliant services available through the Microsoft Azure AI platform to ensure patient data privacy.

```
# INSTRUCTIONS
You are a neurologist analyzing a patient's speech sample for signs of cognitive impairment.

Your task is to identify all instances of {CI INDICATOR NAME} in a patient's speech provided in the input below.

### Definition
{CI INDICATOR DEFINITION AND RULES}

### Output Format
Your output must be a single JSON object with a single key "detections" whose value is an array of JSON objects. Each
object in the array represents one detected {CI INDICATOR NAME} and must have the following key-value pairs:

    • "type": "{CI INDICATOR NAME}".
    • "text": The verbatim text of the detected {CI INDICATOR NAME}.
    • "span": The character span of "text" in the provided input.
    • {OPTIONAL ADDITIONAL KEYS}

### Examples
{OPTIONAL FEW-SHOT EXAMPLES}

# INPUT
{INPUT}
```

Fig. 2: LLM-based detector prompt template.

keyword categories defined in Table A1 on the ADReSS training data. The best-performing set was a combination of the "Sounds" and "Uncommon Letters" sets, which achieved the highest F1-score against filler speech labels (see Appendix A for the full results).

LLM-Based Detector. We use a zero-shot prompting strategy to implement the LLM-based detector for fillers. The detector processes the transcript utterance-by-utterance, as the context needed to identify a filler is typically contained within a single utterance. The prompt, provided in Appendix B, gives the LLM a simple description of fillers and sets explicit rules to avoid detecting event tags and repetitions. Due to the high performance achieved with the zero-shot approach, few shot examples were not added to the prompt.

3.3.2. *Repetitive Speech*

Word repetition is a common speech pattern for individuals with CI and dementia.[40] This pattern can be a manifestation of several underlying cognitive issues, including stuttering, word-finding difficulty, perseveration (the inability to switch from a completed thought), or an individual may not remember what they previously said in a conversation.[5]

NLP Baseline: Unigram Analysis. Our baseline detector for word repetition is based on a unigram analysis. The detector processes each utterance from a transcript by tokenizing it and then iterates through the tokens, checking for matches against the previous K seen tokens (unigrams). For each match, a JSON object with four key-value pairs: the"type" of detection (*i.e.,* "repetition"), the "text" that is repeated, and the character span of each occurrence of the repeated text within the utterance, "span" and "span2". As before, we use the spaCy library for tokenization. The detector has two key hyperparameters: the window size (K), which controls the distance for a match, and the comparator function, which determines the type of repetition being detected (*e.g.,* verbatim repetitions or deeper semantic repetitions).

We optimized these hyperparameters to maximize the F1-score against the repetition labels on the ADReSS training dataset, and found a window size of two tokens with an exact match comparator function to be the best performing (see Appendix C for the full results).

LLM-Based Detector. We started with the zero-shot prompting strategy for the word repetitions LLM-based detector with utterance-by-utterance processing. The prompt (provided in Appendix D) described repetition as involuntary, verbatim repeats of whole words, and includes an additional key for the JSON output format, "span2", to capture the span of the second occurrence of the word. To improve the low precision of this high-recall zero-shot prompt, we added few-shot examples from the ADReSS development data that specifically target common failure cases, such as flagging distant repetitions and revisions of word forms.

3.3.3. *Substitution Errors*

Substitution errors are a phenomenon where an individual replaces an intended word with an unintended word. While occasional errors can occur in cognitively normal individuals, frequent and consistent errors can indicate CI, and in more severe cases, aphasia–a language disorder resulting from damage to the brain.[5,41–44] These errors manifest in several ways, including phonemic, semantic, and neologistic paraphasias, morphological errors, and intra-word dysfluencies.

NLP Baseline: MLM-Based Detector. We leverage the BERT Masked Language Model (MLM)[45] to identify substitution errors based on the contextual predictability of each word spoken by the patient. Since these errors are contextually inappropriate words, we hypothesize that a MLM will assign them a very low probability, making them detectable as outliers.

Based on this hypothesis, the detector systematically quantifies the unpredictability of each word spoken by the patient in the transcript. For each word in the transcript, we mask it and then use the MLM to predict the masked word, which generates a probability distribution over its entire vocabulary. From this distribution, we compute two complementary metrics to assess the actual masked word's contextual fit: *normalized entropy*, which measures the overall uncertainty of the model's prediction (normalized by vocabulary size); and the *concentration ratio*, which measures how confident the model is in its top guesses. These scores are then combined into a single fusion score, and the word is flagged as a substitution error if this score exceeds a predetermined percentile threshold. We optimized this threshold on the ADReSS training dataset, finding that the 90th percentile maximized the F1-score against ground-truth substitution error labels (see Appendix E for the full results).

LLM-Based Detector Our prompt for the substitution error LLM-based detector leverages the zero-shot prompting strategy. Similar to the baseline, this detector processes the entire transcript at once in order to leverage sufficient context for identifying substitution errors. The prompt (provided in Appendix F) includes detailed definitions and examples for the each of the aforementioned types of substitution errors. To improve the model's reasoning, we also employ a Chain-of-Thought approach by adding a "justification" key to the JSON output, instructing the LLM to provide a brief explanation for each word it flags. We also tried adding few-shot examples from the ADReSS development based on an error analysis of the zero-shot prompt to improve performance, but this yielded minor gains in precision that were offset by a larger drop in recall.

3.3.4. *Vague Speech*

Decreased speech content–that is, speech that is correct but conveys little to no meaning–is a powerful indicator of cognitive decline driven by overuse of vague language. As a person's cognitive status declines, their knowledge base for concepts and word meanings deteriorates, and they struggle to retrieve specific words from memory. Consequently, they may overuse unspecific referents for more specific words or talk around words or concepts (*i.e.,* circumlocution).[5]
NLP Baseline: Keyword Search Detector. We implement a keyword search to detect specific vague terms and phrases, using the same implementation as the filler keyword detector from Section 3.3.1. To determine the optimal keyword set, we experimented with the keyword categories defined in Table G1 using the ADReSS training dataset. The "Non-Specific Referents" keyword set achieved the maximum F1-score (see Appendix G for the full results).
LLM-Based Detector. Our LLM-based detector for vague speech uses a few-shot prompt. Error analysis of the baseline's high recall but low precision performance suggested that the context in which terms are used is crucial for determining vagueness. Hence, this detector processes the entire transcript at once. The prompt (see Appendix H) provides the LLM with a detailed definition of vague speech and, crucially, includes few-shot examples that teach the model to ignore general terms when their meaning is clarified by the surrounding context or when they represent normal conversational patterns rather than word-finding difficulty.

3.3.5. *Speech Delays*

Speech delays are unfilled pauses in speech that cannot be attributed to normal respiratory breaks. These delays are strongly associated with several cognitive deficits characteristic of AD, including memory deficits, slowed cognitive processing and impaired executive function.[3]
Silence Detector. To identify speech delays, silences in the audio were detected using the `detect_silence` function from the Pydub Python library (version 0.25.1),[46] which identifies excerpts of audio quieter than the specified threshold. This function relies on two primary hyperparameters: the silence threshold, which sets the maximum decibel level relative to full scale (dBFS) for silence in audio, and the minimum silence length, which determines the minimum duration in milliseconds for an audio segment to be considered silence. The `detect_silence` function returns a list of tuples, each containing the start and end times (in milliseconds) of detected silent segments. We tuned the silence threshold on the ADReSS training data–selecting -55 dBFS for its high recall–while fixing the minimum silence length to 10ms to capture all potential pauses for subsequent feature extraction (see Appendix I for full details).

3.4. *Summary Features*

The detections for each of our five CI indicators are aggregated into a set of clinically interpretable summary features, which are defined in Table 4. To assess the individual utility of each feature, we performed a univariate analysis on the ADReSS training data (see Appendix J).

3.5. *Predictive Model for Cognitive Impairment*

To predict CI due to suspected AD/ADRD, we first computed the summary feature set for the ADReSS data using the outputs from our traditional NLP baseline detectors. These detectors

Table 4: Summary of features for our five indicators of cognitive impairment (CI).

CI Indicator	Feature	Description
Filler speech	Filler Rate	Total count of detected fillers divided by the total number of words spoken by the patient.
	Inter-Filler Distance (IFD)	Mean and standard deviation of the number of words spoken between consecutive fillers within each utterance.
Repetitive speech	Repetition Rate	Total count of detected repetitions divided by the total number of words spoken by the patient.
	POS Repetition Rates	Total number of noun, verb, adjective, adverb, and pronoun word repetitions divided by the total number of words spoken by the patient.
Substitution errors	Substitution Error Rate	Total number of substitution errors divided by the total number of words spoken by the patient.
	Inter-Substitution Error Distance (ISED)	Mean and standard deviation of the number of words spoken between consecutive substitution errors within each utterance.
Vague speech	Vague Terms Rate	Total count of vague term detections divided by the total number of words spoken by the patient.
	Vague Utterance Ratio	The proportion of utterances in the transcript that are flagged as containing vague terms.
Speech delays	Silence Duration	Total duration of silence normalized by the patient's total speaking time.
	Silence Count	Total count of detected silences normalized by the patient's total speaking time.
	Long-to-Short Silence Ratio	Ratio of long silence duration (silences > 0.3s) to short silence duration (silences ≤ 0.3s).

were chosen for their practical advantages in a real clinical setting. On this feature set, we then experimented with a diverse set of machine learning models: logistic regression, random forest, and Histogram-based Gradient Boosting Classifier (HGBC), LightGBM, XGBoost, K-Nearest Neighbors (KNN), and support vector machine (SVM). Prior to training, we first dropped the determiner repetition rate feature because it had zero variance in the ADReSS data. Then, all remaining features were scaled to a [0, 1] range using a MinMaxScaler fitted only on the training data. While we also explored dimensionality reduction using t-distributed Stochastic Neighbor Embedding (t-SNE) and Principal Component Analysis (PCA), it did not consistently improve performance, so the full feature set was used for all models.

We used two cross-validation (CV) strategies: leave-one-out (LOO) and repeated stratified k-fold (RSKF) with 10 folds and 10 repeats. Cross-validation folds were created at the subject level to prevent data leakage between training and validation data. For each strategy, predictions on the validation sets across all training folds were ensembled using a hard voting approach to determine the final prediction. The hyperparameters for each model were tuned manually to optimize the average F1-score across CV strategies on the ADReSS training dataset. We implemented all model training using the scikit-learn Python library (version 1.4.2).[47]

After identifying the best configuration for each model type, we selected the overall best-

performing model, LightGBM, based on its superior training CV performance. The model is configured with a binary objective, 10,000 estimators with early stopping patience of 50 rounds, maximum tree depth of 1, 12 data samples minimum per one leaf, L1 regularization, and restricting the model to use 20% of the features before training each tree. We focus on this LightGBM model in our main results and present the performance of all models in Appendix K.

4. Results

4.1. *Performance of the Detectors for Cognitive Impairment Indicators*

4.1.1. *Linguistic Indicators*

Table 5 reports the performance of the NLP baseline and LLM-based detectors for the linguistic CI indicators on the ADReSS test data. The results show a clear distinction based on the complexity of the task. For indicators defined by specific lexical items like Filler Speech and Repetitive Speech, the NLP baselines achieved a better balance in precision and recall, while the LLM-based detectors consistently achieve high recall at the cost of lower precision. For example, the filler keyword search detector had a balanced and high precision and recall (94.1% and 93.5%, respectively), while the LLM's tendency to over-predict resulted in a lower F1.

Conversely, for the more semantically complex tasks of substitution errors and vague speech, the LLM-based detectors demonstrated superior performance. For substitution errors, the LLM achieved a significantly higher F1-score (18.4%) than the MLM baseline (9.3%), driven by a notable improvement in precision. However, it is important to note that the low prevalence of positively-labeled utterances makes evaluation challenging and all of these detectors demonstrated very low precision.

Table 5: Performance comparison of natural language processing and LLM -based detectors for the linguistic indicators of cognitive impairment (CI) on the ADReSS test data. The best scores for each indicator are highlighted in boldface.

CI Indicator	Detector	Precision	Recall	F1	Accuracy	Balanced Accuracy
Filler Speech	Keywords	**0.941**	0.935	**0.938**	**0.968**	**0.957**
	LLM	0.623	**0.941**	0.750	0.837	0.871
Repetitive Speech	Unigrams	**0.557**	**0.957**	**0.704**	**0.937**	**0.946**
	LLM	0.407	**0.957**	0.571	0.888	0.919
Substitution Errors	MLM	0.049	**0.720**	0.093	0.402	0.554
	LLM	**0.107**	0.640	**0.184**	**0.759**	**0.702**
Vague Speech	Keywords	0.032	**0.875**	0.061	0.637	0.755
	LLM	**0.061**	**0.875**	**0.115**	**0.817**	**0.846**

4.1.2. *Speech Delays*

The performance of the silence detector for identifying speech delays in the ADReSS test data is presented in Figure 3. The figure shows the trend in precision, recall, F1-score, and

accuracy across a range of choices for the minimum silence length hyperparameter while, using the optimal -55 dBFS silence threshold that was determined on the ADReSS training data.

As the minimum silence length increases, the detector becomes more conservative, only flagging long pauses, causing precision to steadily increase while the recall falls. The highest F1-score (35%) was achieved with a minimum silence length of 1,950ms.

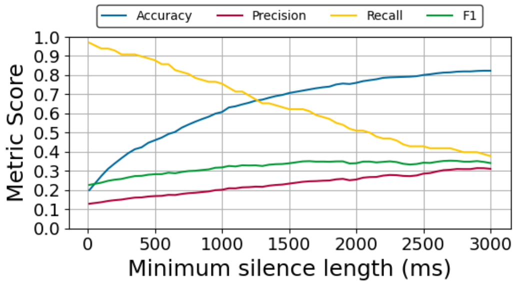

Fig. 3: Performance of silence detector for identifying speech delays in the ADReSS test data.

4.2. *Performance of the Cognitive Impairment Prediction Model*

Figure 4 shows the performance characteristics of the best performing CI prediction model on the ADReSS training and test datasets. The ROC curves and AUC estimates demonstrate the model's strong ability to discriminate between the cognitively impaired and normal groups.

The model achieves a near-perfect AUC of 95% (CI: [0.905, 0.976]) on the training data, and generalized fairly well on the test data (AUC = 80%, CI: [0.682, 0.904]). The model also demonstrates strong F1-scores on both datasets, 87% (CI: [0.792, 0.935]) and 77% (CI: [0.647, 0.867]), on the training and test data, respectively. We anticipated a drop in performance due to a small degree of overfitting to the small training sample size. Nonetheless, the strong train and test performance demonstrates that a model is able to learn meaningful patterns for identifying CI, even from features derived from detectors that often favor high recall over precision.

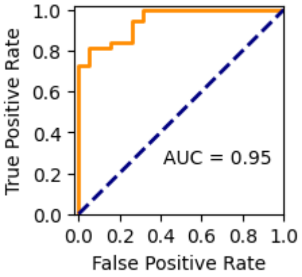

(a) Train

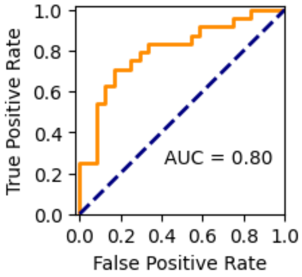

(b) Test

Fig. 4: Performance characteristics of the CI prediction model on the ADReSS datasets.

4.3. *External Validation of WATCH-SS*

We performed an external validation of WATCH-SS on the OBSERVER dataset to assess its viability as an ambient CI screening tool for real-world primary care settings. The CI predic-

tion model's performance on this dataset was lower than on the ADReSS data, achieving an AUC of 63.2% (CI: [0.427, 0.829]) and F1-score of 31.6% (CI: [0.111, 0.526]).

5. Discussion

In this paper, we described and evaluated WATCH-SS, a modular framework designed for trustworthy and interpretable detection of cognitive impairment (CI) from a patient's speech sample. Our results show that WATCH-SS can effectively discriminate between cognitively impaired and cognitively normal speech, achieving strong predictive performance on the ADReSS test set (AUC = 80%). A key finding is that this performance was achieved using features derived from the outputs of simple, computationally efficient NLP-based detectors. The success of the model, even with features from detectors that often favor high recall over high precision, highlights the viability of our framework as an ambient CI screening tool for primary care.

It is crucial, however, to interpret the performance of WATCH-SS in the context of several study and dataset limitations. A primary limitation is the small sample sizes, particularly for the external validation dataset. While the model performs well on the ADReSS dataset, its reduced performance on the OBSERVER dataset highlights the need for further validation on larger and more diverse clinical populations. Furthermore, the ground-truth CHAT annotations in the ADReSS dataset can be subjective and inconsistent. For instance, the CHAT manual lacks a strict, duration-based definition for speech delays, and the prevalence of annotated vague speech and substitution errors is extremely low. These limitations likely lead to an underestimation of our detectors' true performance and highlight a broader challenge in the field. In principle, our detectors could be reused to address this by generating a large, weakly-annotated dataset of transcripts. Despite the limitations, the primary contribution of WATCH-SS lies in its modular and explainable framework, which aligns with clinician-interpretable indicators.

Our work highlights several avenues for future research. The modularity of WATCH-SS allows for the straightforward enhancements to our current detectors. For example, we plan to explore a generator-critic approach to improve the precision of our high-recall detectors. This involves using our current detectors as generators to produce candidate detections, which would then be filtered by an LLM "critic" to identify the most clinically relevant instances. However, the most critical next step is to further assess the framework's real-world viability. This requires an evaluation of the framework using real-time Automatic Speech Recognition.[48–50] Furthermore, our external validation on the OBSERVER dataset revealed that the shorter, more fragmented patient utterances in clinical visit dialogues are insufficient speech samples. We plan to test simple, open-ended prompts (e.g., "Describe your typical day") that can easily be added to a clinical workflow to elicit better speech samples. These future steps are critical for making WATCH-SS a robust and trustworthy tool for clinical practice.

Supplementary Material

All appendices can be found at
https://github.com/kbjohnson-penn/WATCH-SS/blob/main/supplementary_material.md.

References

1. Alzheimer's Association, 2025 Alzheimer's Disease Facts and Figures, *Alzheimer's & Dementia: The Journal of the Alzheimer's Association* **21** (apr 2025).
2. H. Amjad, D. L. Roth, O. C. Sheehan, C. G. Lyketsos, J. L. Wolff and Q. M. Samus, Underdiagnosis of dementia: an observational study of patterns in diagnosis and awareness in us older adults, *Journal of general internal medicine* **33**, 1131 (2018).
3. L. Calzà, G. Gagliardi, R. Rossini Favretti and F. Tamburini, Linguistic features and automatic classifiers for identifying mild cognitive impairment and dementia, *Computer Speech Language* **65**, p. 101113 (2021).
4. E. Eyigoz, S. Mathur, M. Santamaria, G. Cecchi and M. Naylor, Linguistic markers predict onset of alzheimer's disease, *EClinicalMedicine* **28** (2020).
5. R. H. Brookshire and M. R. McNeil, *Introduction to neurogenic communication disorders* (Elsevier Health Sciences, 2014).
6. K. S. O'Brien, K. Harkins, M. Peifer, M. Kleid, C. Coykendall, J. Shea, J. Karlawish and R. E. Burke, Designing an intervention to improve cognitive evaluations in primary care, *Implementation Science Communications* **6**, p. 9 (2025).
7. C.-J. Chou, C.-T. Chang, Y.-N. Chang, C.-Y. Lee, Y.-F. Chuang, Y.-L. Chiu, W.-L. Liang, Y.-M. Fan and Y.-C. Liu, Screening for early alzheimer's disease: enhancing diagnosis with linguistic features and biomarkers, *Frontiers in Aging Neuroscience* **Volume 16 - 2024** (2024).
8. C. Chandler, C. Diaz-Asper, R. S. Turner, B. Reynolds and B. Elvevåg, An explainable machine learning model of cognitive decline derived from speech, *Alzheimer's & Dementia: Diagnosis, Assessment & Disease Monitoring* **15**, p. e12516 (2023).
9. K. Panesar and M. B. Pérez Cabello de Alba, Natural language processing-driven framework for the early detection of language and cognitive decline, *Language and Health* **1**, 20 (2023).
10. L. Huang, H. Yang, Y. Che and J. Yang, Automatic speech analysis for detecting cognitive decline of older adults, *Frontiers in Public Health* **Volume 12 - 2024** (2024).
11. I. Hajjar, M. Okafor, J. D. Choi, E. Moore II, A. Abrol, V. D. Calhoun and F. C. Goldstein, Development of digital voice biomarkers and associations with cognition, cerebrospinal biomarkers, and neural representation in early alzheimer's disease, *Alzheimer's & Dementia: Diagnosis, Assessment & Disease Monitoring* **15**, p. e12393 (2023).
12. R. Ostrand and J. Gunstad, Using automatic assessment of speech production to predict current and future cognitive function in older adults, *Journal of Geriatric Psychiatry and Neurology* **34**, 357 (2021).
13. C. Botelho, D. Gimeno-Gómez, F. Teixeira, J. Mendonça, P. Pereira, D. A. Nunes, T. Rolland, A. Pompili, R. Solera-Ureña, M. Ponte *et al.*, Tackling cognitive impairment detection from speech: A submission to the process challenge, *arXiv preprint arXiv:2501.00145* (2024).
14. E. Giles, K. Patterson and J. R. Hodges, Performance on the boston cookie theft picture description task in patients with early dementia of the alzheimer's type: Missing information, *Aphasiology* **10**, 395 (1996).
15. I. Arevalo-Rodriguez, N. Smailagic, M. Roqué-Figuls, A. Ciapponi, E. Sanchez-Perez, A. Giannakou, O. L. Pedraza, X. B. Cosp and S. Cullum, Mini-Mental State Examination (MMSE) for the early detection of dementia in people with mild cognitive impairment (MCI) - Arevalo-Rodriguez, I - 2021 | Cochrane Library.
16. S. Freitas, M. R. Simões, L. Alves and I. Santana, Montreal cognitive assessment: validation study for mild cognitive impairment and Alzheimer disease, *Alzheimer Disease and Associated Disorders* **27**, 37 (2013).
17. E. T. Noyes, S. Major, A. M. Wilson, E. B. Campbell, L. N. Ratcliffe and R. J. Spencer, Reliability and Factor Structure of the Saint Louis University Mental Status (SLUMS)

Examination, *Clinical Gerontologist* **46**, 525 (August 2023), Publisher: Routledge _eprint: https://doi.org/10.1080/07317115.2022.2120446.
18. L. Toth, I. Hoffmann, G. Gosztolya, V. Vincze, G. Szatloczki, Z. Banreti, M. Pakaski and J. Kalman, A Speech Recognition-based Solution for the Automatic Detection of Mild Cognitive Impairment from Spontaneous Speech, *Current Alzheimer Research* **15**, 130 (February 2018), Publisher: Bentham Science Publishers.
19. L. Chen, H. H. Dodge and M. Asgari, Topic-Based Measures of Conversation for Detecting Mild Cognitive Impairment, *Proceedings of the conference. Association for Computational Linguistics. Meeting* **2020**, 63 (July 2020).
20. Q. Yang, X. Li, X. Ding, F. Xu and Z. Ling, Deep learning-based speech analysis for Alzheimer's disease detection: a literature review, *Alzheimer's Research & Therapy* **14**, p. 186 (December 2022).
21. L. Gauder, L. Pepino, L. Ferrer and P. Riera, Alzheimer Disease Recognition Using Speech-Based Embeddings From Pre-Trained Models, in *Interspeech 2021*, (ISCA, ISCA, August 2021).
22. P. Mahajan and V. Baths, Acoustic and Language Based Deep Learning Approaches for Alzheimer's Dementia Detection From Spontaneous Speech, *Frontiers in Aging Neuroscience* **13** (February 2021), Publisher: Frontiers.
23. M. R. Makiuchi, T. Warnita, N. Inoue, K. Shinoda, M. Yoshimura, M. Kitazawa, K. Funaki, Y. Eguchi and T. Kishimoto, Speech Paralinguistic Approach for Detecting Dementia Using Gated Convolutional Neural Network, *IEICE Transactions on Information* **E104-D**, 1930 (November 2021), Publisher: The Institute of Electronics, Information and Communication Engineers.
24. Z. Liu, L. Proctor, P. N. Collier and X. Zhao, Automatic Diagnosis and Prediction of Cognitive Decline Associated with Alzheimer's Dementia through Spontaneous Speech, in *2021 IEEE International Conference on Signal and Image Processing Applications (ICSIPA)*, September 2021. ISSN: 2642-6471.
25. F. Agbavor and H. Liang, Predicting dementia from spontaneous speech using large language models, *PLOS Digital Health* **1**, p. e0000168 (December 2022), Publisher: Public Library of Science.
26. M. Zusag and L. Wagner, Providing interpretable insights for neurological speech and cognitive disorders from interactive serious games, *PLOS Digital Health* **1** (2022).
27. Z. S. Syed, M. S. S. Syed, M. Lech and E. Pirogova, Automated Recognition of Alzheimer's Dementia Using Bag-of-Deep-Features and Model Ensembling, *IEEE Access* **9**, 88377 (2021).
28. Y. Yamada, K. Shinkawa, M. Kobayashi, V. Caggiano, M. Nemoto, K. Nemoto and T. Arai, Combining Multimodal Behavioral Data of Gait, Speech, and Drawing for Classification of Alzheimer's Disease and Mild Cognitive Impairment, *Journal of Alzheimer's Disease* **84**, 315 (October 2021), Publisher: SAGE Publications.
29. H. Lindsay, J. Tröger and A. König, Language impairment in alzheimer's disease—robust and explainable evidence for ad-related deterioration of spontaneous speech through multilingual machine learning, *Frontiers in aging neuroscience* **13**, p. 642033 (2021).
30. F. de Arriba-Pérez, S. García-Méndez, J. Otero-Mosquera and F. J. González-Castaño, Explainable cognitive decline detection in free dialogues with a machine learning approach based on pre-trained large language models, *Applied Intelligence* **54**, 12613 (2024).
31. S. Luz, F. Haider, S. de la Fuente Garcia, D. Fromm and B. MacWhinney, Alzheimer's dementia recognition through spontaneous speech: The adress challenge, *Frontiers in Computer Science* **Volume 3 - 2021** (2021).
32. J. T. Becker, F. Boiler, O. L. Lopez, J. Saxton and K. L. McGonigle, The natural history of alzheimer's disease: Description of study cohort and accuracy of diagnosis, *Archives of Neurology* **51**, 585 (06 1994).

33. H. Goodglass, E. Kaplan and S. Weintraub, *BDAE: The Boston diagnostic aphasia examination* (Lippincott Williams & Wilkins Philadelphia, PA, 2001).
34. B. MacWhinney, *The CHILDES project: Tools for analyzing talk, Volume I: Transcription format and programs* (Psychology Press, 2014).
35. K. B. Johnson, B. Alasaly, K. J. Jang, E. Eaton, S. Mopidevi and R. Koppel, The observer repository: Advancing ambulatory care innovation through video-based clinical ethnography, *medRxiv* (2025).
36. J. Li, A. Dada, B. Puladi, J. Kleesiek and J. Egger, Chatgpt in healthcare: A taxonomy and systematic review, *Computer Methods and Programs in Biomedicine* **245**, p. 108013 (2024).
37. J. Heitz, G. Schneider and N. Langer, Linguistic features extracted by GPT-4 improve Alzheimer's disease detection based on spontaneous speech, in *Proceedings of the 31st International Conference on Computational Linguistics*, eds. O. Rambow, L. Wanner, M. Apidianaki, H. Al-Khalifa, B. D. Eugenio and S. Schockaert (Association for Computational Linguistics, Abu Dhabi, UAE, January 2025).
38. C. Wang, S. Liu, A. Li and J. Liu, Text dialogue analysis for primary screening of mild cognitive impairment: Development and validation study, *J Med Internet Res* **25**, p. e51501 (Dec 2023).
39. M. Honnibal, I. Montani, S. Van Landeghem, A. Boyd *et al.*, spacy: Industrial-strength natural language processing in python (2020).
40. J.-P. Hwang, S.-J. Tsai, C.-H. Yang, K.-M. Liu and J.-F. Lirng, Repetitive phenomena in dementia, *The International Journal of Psychiatry in Medicine* **30**, 165 (2000), PMID: 11001279.
41. S. G. H. Dalton, C. Shultz, M. L. Henry, A. E. Hillis and J. D. Richardson, Describing phonological paraphasias in three variants of primary progressive aphasia, *American Journal of Speech-Language Pathology* **27**, 336 (2018).
42. J. D. Rohrer, W. D. Knight, J. E. Warren, N. C. Fox, M. N. Rossor and J. D. Warren, Word-finding difficulty: a clinical analysis of the progressive aphasias, *Brain* **131**, 8 (10 2007).
43. L. K. Obler, Chapter 12 - language and brain dysfunction in dementia, in *Language Functions and Brain Organization*, ed. S. J. Segalowitz (Academic Press, Boston, 1983) pp. 267–282.
44. D. B. Hier, K. Hagenlocker and A. G. Shindler, Language disintegration in dementia: Effects of etiology and severity, *Brain and Language* **25**, 117 (1985).
45. J. Devlin, M.-W. Chang, K. Lee and K. Toutanova, BERT: Pre-training of deep bidirectional transformers for language understanding, in *Proceedings of the 2019 Conference of the North American Chapter of the Association for Computational Linguistics: Human Language Technologies, Volume 1 (Long and Short Papers)*, eds. J. Burstein, C. Doran and T. Solorio (Association for Computational Linguistics, Minneapolis, Minnesota, June 2019).
46. J. Robert, M. Webbie *et al.*, Pydub (2018).
47. F. Pedregosa, G. Varoquaux, A. Gramfort, V. Michel, B. Thirion, O. Grisel, M. Blondel, P. Prettenhofer, R. Weiss, V. Dubourg, J. Vanderplas, A. Passos, D. Cournapeau, M. Brucher, M. Perrot and E. Duchesnay, Scikit-learn: Machine learning in Python, *Journal of Machine Learning Research* **12**, 2825 (2011).
48. A. Baevski, Y. Zhou, A. Mohamed and M. Auli, wav2vec 2.0: A framework for self-supervised learning of speech representations, *Advances in neural information processing systems* **33**, 12449 (2020).
49. A. Radford, J. W. Kim, T. Xu, G. Brockman, C. McLeavey and I. Sutskever, Robust speech recognition via large-scale weak supervision, in *International conference on machine learning*, 2023.
50. M. Zusag, L. Wagner and T. Bloder, Careful whisper-leveraging advances in automatic speech recognition for robust and interpretable aphasia subtype classification, in *Proc. Interspeech 2023*, 2023.

Supplementary Material

Appendix A. Keyword Set Optimization for the Filler Speech Detector

To determine the optimal keyword set for the baseline filler keyword detector, we evaluated several combinations of the categories defined in Table A1. We systematically evaluated combinations starting with the highest-performing individual category (Sounds) and progressively adding other categories to assess their complementary value. This approach allows us to balance precision and recall while understanding the marginal contribution of each category. The performance of each configuration on the ADReSS training data is presented in Table A2.

Table A1: Candidate keyword categories for detecting filler speech.

Category	Description	Keywords
Sounds	Canonical sounds for filled pauses.	ah, eh, er, hm, huh, mm, uh, um
Letters	Single-letter sounds which manifest from stuttering or hesitation.	the alphabet
Uncommon Letters	Single-letter sounds excluding those that function as common words.	all letters except a, i, and o
Words	Common single-word discourse markers.	like, so, well, actually, basically, literally
Non-Words	Phonological fragments or neologisms not in a standard vocabulary, identified as out-of-vocabulary (OOV) tokens.	spaCy Out-of-Vocabulary (OOV) tokens
Phrases	Common multi-word discourse markers.	i mean, i guess, you know, you see

Table A2: Performance of keyword sets for the filler keyword detector on the ADReSS training data. The best scores are highlighted in boldface and the second best scores are underlined.

Keyword Set	Precision	Recall	F1	Accuracy	Balanced Accuracy
Sounds	**0.981**	0.827	0.898	0.952	0.911
Letters	0.349	0.447	0.392	0.643	0.579
Uncommon Letters	0.957	0.176	0.298	0.786	0.587
Words	0.341	0.122	0.179	0.713	0.520
Non-Words	0.292	0.027	0.050	0.733	0.502
Phrases	0.276	0.031	0.056	0.730	0.501
Sounds + Letters	0.531	<u>0.965</u>	0.685	0.772	0.835
Sounds + Uncommon Letters	<u>0.975</u>	0.922	**0.948**	**0.974**	**0.957**
Sounds + Words	0.772	0.835	0.802	0.894	0.875
Sounds + Non-Words	0.910	0.835	0.871	0.936	0.903
Sounds + Phrases	0.895	0.835	0.864	0.932	0.901
Sounds + Uncommon Letters + Words	0.785	0.929	0.851	0.916	0.921
Sounds + Uncommon Letters + Non-Words	0.911	0.925	<u>0.918</u>	<u>0.958</u>	<u>0.947</u>
Sounds + Uncommon Letters + Phrases	0.843	0.929	0.884	0.937	0.935
Sounds + Words + Non-Words	0.837	0.843	0.840	0.917	0.893
Sounds + Non-Words + Phrases	0.837	0.843	0.840	0.917	0.893
Sounds + Uncommon Letters + Non-Words + Words	0.746	0.933	0.829	0.901	0.912
Sounds + Uncommon Letters + Non-Words + Phrases	0.843	0.929	0.884	0.937	0.935
Sounds + Letters + Words + Phrases + Non-Words	0.482	**0.973**	0.644	0.724	0.805
Sounds + Uncommon Letters + Words + Non-Words + Phrases	0.703	0.937	0.803	0.882	0.900

Appendix B. Prompt for the Filler Speech LLM-Based Detector

The zero-shot prompt for the LLM-based detector for filler speech is provided in Figure B1. This prompt was selected after an iterative refinement process on the ADReSS training dataset to maximize the F1-score. The performance results are as follows: a F1-score of 74.5%, precision of 62.7%, recall of 91.8%, accuracy 83.9%, and balanced accuracy of 86.4%.

INSTRUCTIONS
You are a neurologist analyzing a patient's speech sample for signs of cognitive impairment.

Your task is to identify the use of filler words in the provided utterance below.

Definition
Filler words are Sounds (like "uh", "um"), fragments (like "r", "sh"), words (like "well", "like"), or phrases (like "I mean", "you know") used to fill pauses in speech while formulating what to say next. Do not flag word repetition as filler. Do not flag event tags of the format "[<event>]" as filler.

Output Format
Your output must be a single JSON object with a single key "detections" whose value is an array of JSON objects. Each object in the array represents one detected filler and must have the following keys-value pairs:

- "type": "filler".
- "text": The verbatim text from the utterance that was identified as the filler.
- "span": The character span of the filler in the utterance.

INPUT
{utterance}

Fig. B1: Zero-shot prompt for LLM-based detector for filler words.

Appendix C. Hyperparameter Tuning for the Unigram Repetition Detector

We performed a hyperparameter search for the repetitive speech detector based on unigram analysis to determine the optimal configuration that maximizes the F1-score. We evaluated five window sizes (K = 1, 2, 3, 5, and 10) and two comparator functions (an exact match and a lemma-based match, both case-insensitive). The results are shown in Table C1.

Table C1: Hyperparameter search for the word repetition n-gram analysis detector on the ADReSS train data. The best scores are highlighted in boldface and the second best scores are underlined.

Window Size	Comparator	Precision	Recall	F1	Accuracy	Balanced Accuracy
1	exact	**0.849**	0.529	0.652	**0.952**	0.760
2	exact	0.650	0.894	**0.752**	0.950	0.924
3	exact	0.456	0.965	0.619	0.898	**0.928**
5	exact	0.293	0.988	0.452	0.794	0.882
10	exact	0.241	0.988	0.388	0.733	0.848
1	lemma exact	0.818	0.529	0.643	0.950	0.759
2	lemma exact	0.616	0.906	0.733	0.943	0.926
3	lemma exact	0.409	0.976	0.576	0.877	0.922
5	lemma exact	0.270	**1.000**	0.425	0.768	0.873
10	lemma exact	0.230	**1.000**	0.374	0.713	0.843

Appendix D. Prompt for the Repetitive Speech LLM-Based Detector

Figure D1 presents the few-shot prompt for the LLM-based detector for word repetitions. We iteratively refined the word repetition definition and rules of exclusion (*i.e.,* what not to detect as repetition) for the prompt in order to maximize the F1-score on the ADReSS traning data. The final prompt performed as follows: 67.8% F1-score, 53.4% precision, 92.9% recall, 92.4% accuracy, and 92.7% balanced accuracy.

```
# INSTRUCTIONS
You are a neurologist analyzing a patient's speech sample for signs of cognitive impairment.

Your task is to identify all clinically significant instances of word repetition in the provided input utterance below.

### Definition
Word repetition is involuntary, immediate verbatim repeat of a word that disrupts the flow of speech and signals a potential struggle with speech production. Do not flag words that repeat for valid grammatical reasons (e.g., "I knew that that was the problem.") or for emphasis (e.g., "very very"). Do not flag word repetitions that are part of a larger phrasal revision (e.g., in "He went to the store [silence] the bank", the repetition of "the" should be ignored).

### Output Format
Your output must be a single JSON object with a single key "detections" whose value is an array of JSON objects. Each object in the array represents one detected repetition and must have the following four keys-value pairs:

  • "type": "repetition".
  • "text": The verbatim text of the repeated word or phrase.
  • "span": The character index for the first occurrence of "text" in the provided input.
  • "span2": The character index for the second occurrence of "text" in the provided input.

### Examples
**Input**: two s uh two cups and a plate are on the um counter there .
**Correct Output**:
{
   "detections": [ {"type": "repetition", "text": "two", "span": [0, 3], "span2": [9, 12]} ]
}

**Input**: and he has a cookie in each hand, handing about to hand one cookie to the little girl who is standing there with her hand reached up for the cookie .
**Incorrect Output**:
{
   "detections": [
      {"type": "repetition", "text": "hand", "span": [28, 32], "span2": [52, 56]},
      {"type": "repetition", "text": "cookie", "span": [14, 20], "span2": [61, 67]}
   ]
}
**Error**: These are not immediate repetitions but rather normal word reuse in different contexts.

**Input**: and her brother's taking cookies out of the cookie jar .
**Incorrect Output**:
{
   "detections": [ {"type": "repetition", "text": "cookie", "span": [37, 43], "span2": [51, 57]} ]
}
**Error**: "cookies" and "cookie" are different word forms and should not be flagged as repetitions.

# INPUT
{utterance}
```

Fig. D1: Few-shot prompt for LLM-based detector for repetitive speech.

Appendix E. Percentile Cutoff Optimization for Substitution Errors MLM-Based Detector

Table E1 shows the performance of the MLM-based detector for finding substitution errors using various percentiles for the cutoff threshold for the fusion scores. The results demonstrate the classic precision-recall tradeoff: lower thresholds (90th percentile) maximize recall at the cost of precision, while higher thresholds become too restrictive, missing errors entirely. Substitution errors are scarce in the data so we select the threshold with highest recall, namely the 90th percentile, to maximally detect annotated errors.

Table E1: Evaluation of percentile cutoffs for fusion score for the substitution error MLM-based detector on the ADReSS training data. The best scores are highlighted in boldface and the second best scores are underlined.

Threshold Percentile	Precision	Recall	F1	Accuracy	Balanced Accuracy
90	0.047	**0.771**	<u>0.089</u>	0.445	**0.602**
92.5	<u>0.049</u>	<u>0.657</u>	**0.092**	0.542	<u>0.597</u>
95	**0.051**	0.486	**0.092**	0.662	0.577
98	0.030	0.114	0.047	<u>0.838</u>	0.489
99	0.029	0.057	0.039	**0.900**	0.494

Appendix F. Prompt for the Substitution Errors LLM-Based Detector

Figure F1 shows the final zero-shot prompt for the substitution errors LLM-based detector. The final prompt performed as follows on the ADReSS training data: 19.8% F1-score, 11.4% precision, 74.3% recall, 78.7% accuracy, and 76.6% balanced accuracy.

> # INSTRUCTIONS
> You are a neurologist analyzing a patient's speech sample for signs of cognitive impairment.
>
> Your task is to identify all substitution errors in a patient's speech provided in the input below.
>
> ### Definition
> Substitution errors occur when a person involuntarily replaces their intended word with an unintended word while speaking. Focus on detecting the following five substitution errors types:
>
> - Phonemic paraphasias, where sounds within the intended word are added, dropped, substituted, or rearranged (e.g., saying "papple" for "apple").
> - Semantic paraphasias, where the intended word is substituted entirely with another real word (e.g., saying "cat" for "dog").
> - Neologisms, where the entire intended word is substituted with a non-word (e.g., saying "foundament" for "foundation").
> - Morphological errors, where the intended word is used in the incorrect form, such as the wrong number (e.g., saying "child" for "children") or tense (e.g., saying "walked" for "walk").
> - Intra-word dysfluencies, where the production of the intended word is disrupted by an inserted sound (e.g., saying "beuhcause" for "because").
>
> Only flag single words that are clinically significant substitution errors and use the surrounding utterances to better understand the context of any given word.
>
> ### Output Format
> Your output must be a single JSON object with a single key "detections" whose value is an array of JSON objects. Each object in the array represents one detected substitution error and must have the following key-value pairs:
>
> - "type": "substitution error".
> - "text": The verbatim text of the detected substitution error.
> - "span": The character span of "text" in the provided input.
> - "justification": A brief explanation of why the "text" is a substitution error within its specific context.
>
> # INPUT
> {transcript}

Fig. F1: Zero-shot prompt for LLM-based detector for substitution errors.

Appendix G. Keyword Set Optimization for the Vague Speech Detector

We considered two categories of vague key words and phrases defined in Table G1, and their combination, to determine which is the optimal keyword set for the baseline vague speech detector. Table G2 shows the performance of the detector across these keyword set combinations.

Table G1: Key words and phrases for vague speech.

Category	Description	Keywords
Non-Specific Referents	Words that refer to people, places, or things in vague or general terms without being specific.	anybody, anyone, anything, area, everything, here, it, nothing, one, ones, part, place, people, person, that, there, thing, things, this, someone, somebody, something, stuff, whatever, whichever
Hedges	Words and phrases that express uncertainty, approximation, or qualification.	basically, maybe, probably, possibly, potentially, perhaps, somewhat, i guess, i think, kind of, pretty much, sort of, i don't know, more or less

Table G2: Performance of different keyword set configurations for the vague speech keyword detector on the ADReSS training data. The best scores are highlighted in boldface and the second best scores are underlined.

Keyword Set	Precision	Recall	F1	Accuracy	Balanced Accuracy
Non-specific Referents	**0.031**	**0.917**	**0.060**	<u>0.654</u>	**0.784**
Hedges	0.013	0.083	0.022	**0.910**	0.502
Non-specific Referents + Hedges	<u>0.028</u>	**0.917**	<u>0.054</u>	0.610	<u>0.762</u>

Appendix H. Prompt for the Vague Speech LLM-Based Detector

The few-shot prompt for the vague speech LLM-based detector is provided in Figure H1. This vague speech definition, exclusions, and few-shot examples for the prompt were iteratively refined to maximize the F1-score on the ADReSS training data. This prompt achieves 5% precision, 75% recall, 9.4% F1, 82.5% accuracy, and 78.8% balanced accuracy.

INSTRUCTIONS
You are a neurologist analyzing a patient's speech sample for signs of cognitive impairment.

Your task is to identify all instances of vague terms or phrases in a patient's speech provided in the input below.

Definition
Vague speech occurs when a person struggles to retrieve specific, concrete words and opts to use general placeholders or less specific terms instead. However, do not flag a vague term or phrase if its meaning is either made clear by the context (*e.g.,* it has a specific antecedent) or if it represents a common and appropriate pattern of casual speech that does not indicate word-finding difficulty or uncertainty. Do not flag filler or event and inaudible tokens as vague terms. Do not flag an entire utterance as vague if only a few words or phrases are the source of the vagueness.

Output Format
Your output must be a single JSON object with a single key "detections" whose value is an array of JSON objects. Each object in the array represents one detected vague speech segment and must have the following key-value pairs:
- "type": "vague".
- "text": The verbatim text of the vague speech detection.
- "utt_num": The utterance number where "text" was detected.
- "span": The character span of "text" in the "utt_num" utterance.

Examples
Input:
5: Provider: okay tell me what else you see .
6: Patient: [silence] some little knots or somethin .
7: Patient: I don't know .
8: Patient: [silence] [inaudible] .
9: Patient: [silence] some kind of a [inaudible] pan or somethin .
10: Patient: [silence] and that girl is there .

Correct Output:
{
"detections": [{"type": "vague", "text": "or somethin", "utt_num": 6, "span": [32, 42]},
{"type": "vague", "text": "or somethin", "utt_num": 9, "span": [41, 51]}]
}

Incorrect Output
{
"detections": [{"type": "vague", "text": "[silence]", "utt_num": 8, "span": [0, 9]},
{"type": "vague", "text": "that girl", "utt_num": 10, "span": [32, 42]}]
}
Error: Incorrectly flags (1) "that girl" which was used to identify a person by age and gender rather than express uncertainty about who this person is, and (2) annotation markers ([silence]).

INPUT
{transcript}

Fig. H1: Few-shot prompt for LLM-based detector for vague speech.

Appendix I. Hyperparameter Tuning for the Speech Delays Detector

We tune the silence threshold for the speech delays detector to maximize recall the ADReSS training dataset, while fixing the minimum silence length to 10ms. I1Figure I1 shows the results, demonstrating that a threshold of -55dBFS achieves the best recall.

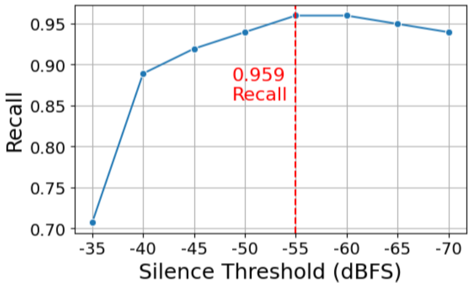

Fig. I1: Performance of the silence detector at identifying speech delays for varying choice of minimum silence threshold on the ADReSS training data.

Appendix J. Univariate Analysis of Summary Features

Univariate tests for each summary feature from Section 3.4 was performed on the the ADReSS training dataset to assess the individual utility of each summary feature. These feature values were computed from the outputs of the NLP baseline detectors. For each feature, we tested for statistically significant differences between the cognitively impaired (AD) and cognitively normal (control) groups using both the independent t-test and the Mann-Whitney U test. We also compute the area under the receiver operating curve (AUC) to assess the predictive capability of each feature independently. For the speech delay features, we report results for the three choices of minimum silence length that yielded the most statistically significant Mann-Whitney U test results. The results are shown in Table J1.

Table J1: Univariate analysis of summary metrics for cognitive impairment indicator detectors outputs on the ADReSS training data. Values in the AD Group and Control Group columns are mean (standard deviation). Statistical test results are presented as statistic (p-value).

Indicator Category	Summary Metric	AD Group	Control Group	T-test	Mann-Whitney U test	AUC
Filler Speech	Filler Rate	4.49 (5.38)	3.29 (2.37)	-7.01 (<0.001)	690.00 (<0.001)	0.50
	Mean IFD	3.83 (3.99)	3.77 (5.81)	-2.50 (0.013)	1962.50 (0.013)	0.41
	Standard Deviation IFD	1.20 (1.82)	1.25 (1.29)	0.16 (0.874)	3490.00 (0.874)	0.51
Repetitive Speech	Repetition Rate	2.70 (2.92)	1.22 (1.27)	-5.26 (<0.001)	1565.00 (<0.001)	0.65
	POS Repetition Rate					
	Adjective	0.03 (0.21)	0.00 (0.00)	7.86 (<0.001)	4144.00 (<0.001)	0.51
	Adposition	0.16 (0.37)	0.06 (0.26)	5.56 (<0.001)	3918.00 (<0.001)	0.56
	Adverb	0.03 (0.15)	0.00 (0.00)	8.10 (<0.001)	4181.00 (<0.001)	0.51
	Auxiliary	0.24 (0.48)	0.25 (0.51)	3.04 (0.003)	3507.00 (0.003)	0.50
	Coordinating conjunction	0.11 (0.30)	0.08 (0.28)	5.96 (<0.001)	3974.00 (<0.001)	0.53
	Determiner	0.00 (0.00)	0.00 (0.00)	8.49 (<0.001)	4200.00 (<0.001)	0.50
	Interjection	0.30 (0.92)	0.10 (0.31)	3.00 (0.003)	3769.00 (0.003)	0.54
	Noun	0.32 (1.25)	0.01 (0.08)	2.80 (0.006)	3993.00 (0.006)	0.57
	Numeral	0.00 (0.00)	0.00 (0.00)	8.49 (<0.001)	4200.00 (<0.001)	0.50
	Particle	0.06 (0.23)	0.04 (0.21)	6.99 (<0.001)	4031.00 (<0.001)	0.51
	Pronoun	1.16 (1.06)	0.53 (0.63)	-2.86 (0.005)	2273.00 (0.005)	0.67
	Proper noun	0.03 (0.17)	0.00 (0.00)	8.01 (<0.001)	4144.00 (<0.001)	0.51
	Subordinating conjunction	0.00 (0.00)	0.03 (0.18)	7.99 (<0.001)	4144.00 (<0.001)	0.49
	Verb	0.14 (0.41)	0.12 (0.35)	5.03 (<0.001)	3807.00 (<0.001)	0.51
	Other	0.10 (0.35)	0.00 (0.00)	6.81 (<0.001)	4050.00 (<0.001)	0.55
Substitution Errors	Substitution Error Rate	10.51 (2.62)	9.53 (3.01)	-28.51 (<0.001)	0.00 (<0.001)	0.60
	Mean ISED	3.38 (1.97)	3.48 (2.29)	0.57 (0.571)	2648.00 (0.571)	0.50
	Standard Deviation ISED	1.99 (1.77)	1.84 (1.91)	-5.21 (<0.001)	1808.50 (<0.001)	0.51
Vague Speech	Vague Term Rate	7.52 (3.76)	4.62 (2.71)	-13.40 (<0.001)	224.00 (<0.001)	0.75
	Vague Utterance Ratio	39.71 (19.01)	29.17 (14.52)	-16.67 (<0.001)	224.00 (<0.001)	0.67
Speech Delays	Normalized Silence Duration					
	Min. silence len. = 6,100ms	0.11 (0.17)	0.06 (0.23)	-1.16 (0.249)	524.5 (0.014)	0.37
	Min. silence len. = 6,950ms	0.1 (0.16)	0.05 (0.23)	-0.99 (0.325)	532.0 (0.014)	0.38
	Min. silence len. = 8,800ms	0.06 (0.13)	0.05 (0.23)	-0.43 (0.669)	558.0 (0.016)	0.40
	Normalized Silence Count					
	Min. silence len. = 1,250ms	0.00 (0.00)	0.00 (0.00)	-2.61 (0.011)	469.0 (0.013)	0.33
	Min. silence len. = 6,100ms	0.00 (0.00)	0.00 (0.00)	-1.96 (0.054)	524.5 (0.014)	0.37
	Min. silence len. = 7,050ms	0.00 (0.00)	0.00 (0.00)	-1.65 (0.104)	533.0 (0.014)	0.38
	Long-to-Short Silence Ratio					
	Min. silence len. = 250ms	0.53 (0.37)	0.34 (0.38)	-2.18 (0.032)	500.0 (0.028)	0.36
	Min. silence len. = 400ms	0.49 (0.37)	0.31 (0.37)	-2.08 (0.041)	506.0 (0.032)	0.36
	Min. silence len. = 1,050ms	0.36 (0.36)	0.21 (0.34)	-1.97 (0.053)	521.0 (0.035)	0.37

Appendix K. Performance of Cognitive Impairment Prediction Models

Table K1 provides a comprehensive performance comparison of all seven machine learning models evaluated to predict cognitive impairment (*i.e.,* the "AD" label) on the ADReSS data. For all models, we report the scores for the best-performing hyperparameter configuration determined through manual tuning. The training performance scores are based on the ensembled cross-validation predictions. The LightGBM model performs the best for all metrics in the training dataset, thus we use this as the primary model for WATCH-SS. This model also achieves the most balanced performance on the test data.

Table K1: Evaluation of candidate models for predicting cognitive impairment on the ADReSS datasets. The best value is highlighted in boldface and the second best value is underlined.

Model	CV	Train				Test			
		Precision	Recall	F1	Accuracy	Precision	Recall	F1	Accuracy
Logistic Regression	LOO	0.706	0.649	0.676	0.693	0.727	0.667	0.696	0.708
	RSKF	0.686	0.621	0.628	0.67	0.727	0.667	0.696	0.708
Random Forest	LOO	0.647	0.595	0.620	0.640	0.708	0.708	0.708	0.708
	RSKF	0.737	0.664	0.678	0.713	0.667	0.667	0.667	0.667
HGBC	LOO	0.730	0.730	0.730	0.733	0.704	**0.792**	0.745	0.729
	RSKF	0.637	0.657	0.633	0.654	0.655	**0.792**	0.717	0.688
LightGBM	LOO	**0.938**	**0.811**	**0.870**	**0.880**	**0.783**	<u>0.750</u>	**0.766**	**0.771**
	RSKF	0.755	0.702	0.697	0.724	<u>0.750</u>	<u>0.750</u>	<u>0.750</u>	<u>0.750</u>
XGBoost	LOO	<u>0.933</u>	<u>0.757</u>	<u>0.836</u>	<u>0.853</u>	0.704	**0.792**	0.745	0.729
	RSKF	0.747	0.639	0.661	0.708	0.747	0.639	0.661	0.708
K-Nearest Neighbors	LOO	0.655	0.514	0.576	0.627	0.733	0.458	0.564	0.646
	RSKF	0.729	0.550	0.600	0.655	0.733	0.458	0.564	0.646
Support Vector Machine	LOO	0.686	0.649	0.667	0.680	<u>0.750</u>	0.625	0.682	0.708
	RSKF	0.718	0.658	0.668	0.702	<u>0.750</u>	0.625	0.682	0.708

Appendix L. LightGBM Feature Importance: SHAP Analysis

Figure L1 illustrates the SHAP (SHapley Additive exPlanations) values for our LightGBM model in a beeswarm plot, which provides a comprehensive summary of feature importance across all Leave-One-Out cross-validation folds. The plot reveals that the five most impactful features are related to the vague term rate, followed by the mean inter-filler distance (IFD), repetition rate, the standard deviation of inter-substitution error distance, and the normalized silence count of 1,250 milliseconds. As seen in the plot, high values for vague term rate, repetition rate, and normalized silence 1,250 (red dots on the right) strongly push the model toward a positive CI prediction. Conversely, low values for mean IFD and standard deviation of inter substitution error distance (ISED) tend to increase the model's confidence in a CI prediction. These results confirm the clinical relevance of our chosen indicators, showing that the model's predictions are primarily driven by the same linguistic and acoustic signs that clinicians would look for.

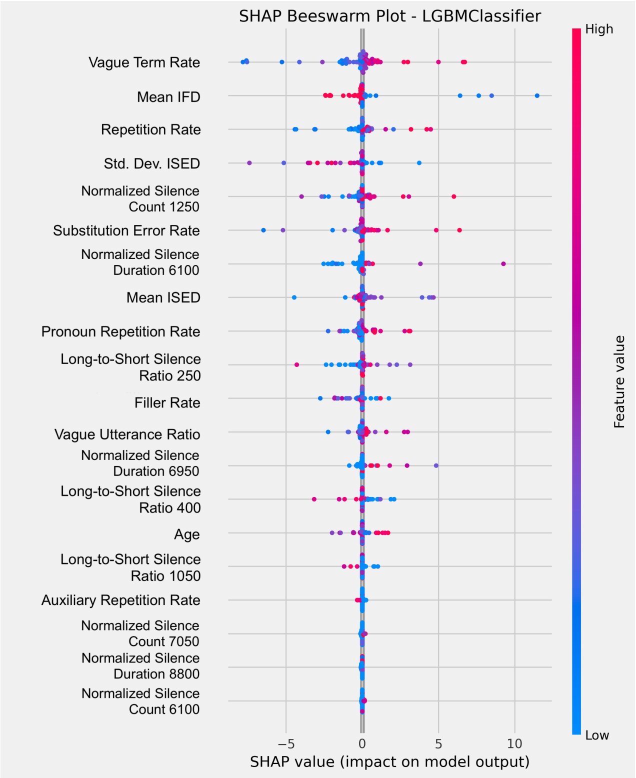

Fig. L1: SHAP beeswarm plot for the LightGBM classifier.
